# Injuries to the Base of the Brain

**Published:** 1917-06

**Authors:** M. M. Walker

**Affiliations:** Wichita Falls, Texas


### INJURIES TO THE BASE OF THE BRAIN.

BY DR. M. M. WALKER, OF WICHITA FALLS, TEXAS.

Injuries to the base of the brain are usually caused by direct violence, and are usually attended by a fracture of the skull. However, frequently certain symptoms will appear following an injury when no fracture or other external evidence of injury can be seen. These symptoms are varied and are perplexing to the surgeon, and an intimate knowledge of the anatomy and physiology of the structure forming the base of the brain is necessary to make a diagnosis. These symptoms, when not attended by a fracture, are usually caused by a laceration of the tissues in this region due to a concussion or to a hemorrhage, causing a deposit or a clot in one of the spaces of the brain in this region, usually in the fourth ventricle. The symptoms of these injuries are usually characterized by the absence of local symptoms, usually having no local pain, if pain at all, and most frequently the special senses are not interfered with.

The symptoms produced are general in character, stupor and coma being early symptoms which disappear after a few hours. Nausea and vomiting are other important symptoms. However, these symptoms can be caused from injuries to other localities in the brain. General convulsions is another important symptom. This symptom, when caused by injury to the cortex, is readily controlled. Therefore, when presistent, it is usually due to an injury to the pons.

The secretion of an excessive amount of urine with sugar is a symptom which is always present. The continued vertigo following a head injury will usually lead us to believe that there has been a disturbance of the mechanism which presides over equilibrium, this mechanism being located in the base of the brain. A rapid rise in the pulse on exertion, when taken in conjunction with other symptoms, is an aid to diagnosis of an injury of this region, as this symptom can be caused by a reflex action through the pneumogastric nerve. I consider a rapid and distressed respiration on exertion to be one of the most characteristic symptoms of an injury to this region.

One point that I tried to make in the discussion of this subject is the difficulty of making a diagnosis in these cases. A diagnosis can only be made after careful study of many obscure symptoms. The treatment in these cases is disappointing and is expectant, and beyond rest in bed is practically nil, as surgical interference is extremely hazardous.

